# Associations between Cognition, Gender and Monocyte Activation among HIV Infected Individuals in Nigeria

**DOI:** 10.1371/journal.pone.0147182

**Published:** 2016-02-01

**Authors:** Walter Royal, Mariana Cherner, Tricia H. Burdo, Anya Umlauf, Scott L. Letendre, Jibreel Jumare, Alash’le Abimiku, Peter Alabi, Nura Alkali, Sunday Bwala, Kanayo Okwuasaba, Lindsay M. Eyzaguirre, Christopher Akolo, Ming Guo, Kenneth C. Williams, William A. Blattner

**Affiliations:** 1 Department of Neurology, University of Maryland, School of Medicine, Baltimore, Maryland, United States of America; 2 HIV Neurobehavioral Research Center, University of California San Diego, School of Medicine, San Diego, California, United States of America; 3 Department of Biology, Boston College, Chestnut Hill, Massachusetts, United States of America; 4 Institute for Human Virology, University of Maryland, School of Medicine, Baltimore, Maryland, United States of America; 5 Institute for Human Virology-Nigeria, Abuja, Nigeria; 6 University of Abuja Teaching Hospital, Gwagwalada, Abuja, Nigeria; 7 Abubakar Tafawa Balewa University Teaching Hospital, Bauchi, Nigeria; 8 National Hospital, Abuja, Nigeria; University of Nebraska Medical Center, UNITED STATES

## Abstract

The potential role of gender in the occurrence of HIV-related neurocognitive impairment (NCI) and associations with markers of HIV-related immune activity has not been previously examined. In this study 149 antiretroviral-naïve seropositive subjects in Nigeria (SP, 92 women and 57 men) and 58 seronegative (SN, 38 women and 20 men) were administered neuropsychological testing that assessed 7 ability domains. From the neuropsychological test scores was calculated a global deficit score (GDS), a measure of overall NCI. Percentages of circulating monocytes and plasma HIV RNA, soluble CD163 and soluble CD14 levels were also assessed. HIV SP women were found to be younger, more educated and had higher CD4+ T cell counts and borderline higher viral load measures than SP men. On the neuropsychological testing, SP women were more impaired in speed of information processing and verbal fluency and had a higher mean GDS than SN women. Compared to SP men, SP women were also more impaired in speed of information processing and verbal fluency as well as on tests of learning and memory. Numbers of circulating monocytes and plasma sCD14 and sCD163 levels were significantly higher for all SP versus all SN individuals and were also higher for SP women and for SP men versus their SN counterparts. Among SP women, soluble CD14 levels were slightly higher than for SP men, and SP women had higher viral load measurements and were more likely to have detectable virus than SP men. Higher sCD14 levels among SP women correlated with more severe global impairment, and higher viral load measurements correlated with higher monocyte numbers and sCD14 and sCD14 levels, associations that were not observed for SP men. These studies suggest that the risk of developing NCI differ for HIV infected women and men in Nigeria and, for women, may be linked to effects from higher plasma levels of HIV driving activation of circulating monocytes.

## Introduction

Neurocognitive impairment is a common consequence of HIV-1 infection and has been increasing as infected individuals survive longer with treatment with antiretroviral therapy [1,2]. Clinically, the determination of NCI in HIV-infected individuals is made based on performance on detailed neuropsychological testing combined with evidence of impairment in functioning in activities in everyday living. In previous studies in Nigeria, evidence of NCI among HIV infected individuals has been documented using both screening tests and detailed neuropsychological test batteries [3–6]. These studies have demonstrated the presence of patterns of impairment that are similar to what has been described in other countries in Africa as well as in more developed areas of the world. This is important because it suggests that information which is obtained from such assessments can be applied to determine whether factors that have been previously linked with an increased risk for developing NCI may also play a role in the development of the disorder among HIV-infected individuals in Nigeria.

Among the demographic factors that have been studied for possible associations with HIV disease progression in the development of NCI is gender. In a small study performed in Zambia in which HIV seronegative (SN) and seropositive (SP) individuals were administered a detailed neuropsychological battery, SP women were found to perform worse in a number of cognitive domains than their SN counterparts [7]. Findings from other cohorts, however, have differed as to whether gender may impact neuropsychological performance, with most studies either not demonstrating an effect [8–10] or with the differences being explained by such factors as level of education or substance use [11]. In recent years it has been demonstrated that neurocognitive impairment in HIV infection correlates with levels of markers of mononuclear phagocyte activation, in particular, levels of soluble CD14 (sCD14) and soluble CD163 (sCD163) [12–17]. Both markers are proteins that are expressed on the cell surface and secreted with cellular activation, either from the membrane surface from cytoplasmic vesicles. In this report, we describe studies in which HIV SP Nigerian women were found to perform worse on a neuropsychological battery than their male SP counterparts. Such differences appear to be linked to differences in the pattern of expression of specific biomarkers of HIV disease activity.

## Materials and Methods

### Study recruitment and participants

Study participants were consecutively recruited form HIV testing and counseling centers located at the National Hospital and the University of Abuja Teaching Hospital, both in Abuja, Nigeria, the nation’s capital city. These hospitals are within a network of sites supported by the Institute of Human Virology, Nigeria (IHVN) with funds from the U.S. President’s Emergency Plan for AIDS Relief (PEPFAR) program. All individuals were ≥18 years of age, able to converse in English and were antiretroviral naïve. Information related to demographics, clinical symptoms and medical, psychiatric and substance use history was obtained through the administration of standardized questionnaires. Participants were excluded who had active tuberculosis, syphilis or other CNS infection or evidence of the presence of a clinical problem that could impair their ability to participate in the testing, including other active CNS or systemic disease, a history of significant head trauma, a history of alcohol abuse, use of other mind-altering substances, or, if there was a history of substance use, evidence of such use on urine toxicology screening. There was also no previous diagnosis of a learning disability or psychiatric disorder or other disorders associated with the presence of focal neurological signs or deficits. Following enrollment the participants underwent a thorough general medical assessment and neuropsychological testing. They also underwent phlebotomy and subsequent determination of HIV-1 serological status, viral load (limit of detection 400 copies/ml) and measurement of CD4+ T cell count performed at the Institute of Human Virology, Nigeria-supported Research Laboratory located in Asokoro, Abuja. Written consent was obtained from all study participants. Using information obtained from the clinical history and medical examination, the clinical stage of the participants was determined using the World Health Organization clinical classification for established HIV infection (stage 1 = asymptomatic infection; 2 = mild symptoms; 3 = advanced symptoms; 4 = severe symptoms) [18]. All study procedures were approved by the Institutional Review Boards of the University of Maryland, Baltimore, the National Hospital in Abuja, Nigeria and University of Abuja Teaching Hospital in Gwagwalada, Nigeria and by the Nigerian National Health Research Ethics Committee.

### Neuropsychological assessment

A detailed standardized neuropsychological battery was administered to all study participants by an examiner who was blinded to the HIV serological status of the participant. The following ability domains, followed by the individual tests within the domains in parentheses, were examined: speed of information processing (WAIS-III Digit Symbol, WAIS-III Symbol Search, Color Trails Test 1 and Trail Making Test A); attention/working memory (Paced Auditory Serial Addition Task, and WMS-III Spatial Span); executive functions (Color Trails Test 2 and Stroop Color and Word Test); learning (Hopkins Verbal Learning Test—Revised (HVLT-R) total learning and the Brief Visuospatial Memory Test—Revised (BVMT-R) total learning); memory (HVLT-R delayed recall; BVMT-R delayed recall); verbal fluency (Letter (Word Sound) Fluency; Category Fluency: Nouns (animals) and Category Fluency: Verbs (actions)); motor speed and dexterity (Grooved Pegboard Test-Dominant Hand, Finger Tapping Test-Dominant Hand and Timed Gait), and screening for effort (Hiscock Digit Memory Test). Raw scores from the neuropsychological tests were converted to scaled scores and the scaled scores were used to generate standardized scores (T-scores) that were adjusted for age, education and gender. Individual test T-scores were then used to calculate a deficit score, which were averaged for tests within each cognitive domain to generate a domain deficit score, which were averaged across all domains to calculate a global deficit score (GDS) [19]. In addition to recruiting only English speakers, to minimize the potential impact of language or cultural differences on performance on the testing, words were eliminated from verbal tests that were likely to be unfamiliar to individuals from the region and, after appropriate pilot testing, were replaced by more familiar terms. Information regarding the presence and severity of symptoms of depression were collected using the Beck Depression Inventory-2^nd^ Edition (BDI-II) [20].

### Blood sample preparation and storage

Peripheral blood samples were collected in citrate tubes and cells and plasma was separated on Ficoll gradients as previously described [21]. Aliquots of plasma were stored at -20 degrees until analyzed.

### Enzyme-linked Immunosorbent Assays

Commercially available kits were used to measure plasma soluble CD163 (sCD163; Trillium Diagnostics) and soluble CD14 (sCD14; R&D Systems) levels, which were measured by enzyme-linked Immunosorbent assays according the manufacturers’ directions and as previously described [21]. The limit of detection for the kits was <0.23 ng/ml for sCD163 and 62.5 pg/ml for sCD14.

### Statistical Analysis

Demographic, clinical, immunological and virological data and neuropsychological test T scores, deficit scores and global deficit scores were compared for SP and SN participants and by gender using t-tests. Comparisons involving individuals categorized by both HIV serological status and gender were performed using analysis of variance (ANOVA) with correction for multiple comparisons using the Tukey multiple comparisons test. Rates of impairment and select categorical demographic data were examined using the Chi-square test or Fisher’s Exact Test, as appropriate. Associations between GDS and other variables were examined by calculating Spearman’s rank correlation coefficients. Percent monocyte values and viral load measurements were log transformed to achieve normality for these data. Statistical significance is indicated by a p-value <0.05. Statistical analyses were performed using SAS Enterprise Guide (SAS Institute, Inc.) and GraphPad Prism 6 for Windows (GraphPad Software, Inc.).

## Results

### Comparison of Demographic and General Clinical Characteristics

Comparison of the demographic and clinical characteristics for the 58 SN and 149 SP participants showed that SN subjects were younger and had more years of education than SP individuals (Table 1). BDI-II scores were lower for SN than for SP subjects. The percentage of males and females were similar for the SN and SP participant groups. However, SN men were significantly younger than SP men, and SP women were significantly younger than SP men. SN women and men were more educated than their SP counterparts, and there was no difference in the level of education for SN women versus SN men and for SP women versus SP men. Mean depression scores were higher for SP men than for their SN counterparts and higher for SN women than for SN men. CD4+ T cell counts were lower for SP than for SN participants and lower for SN and SP men as compared to their female counterparts. Among HIV-infected individuals, HIV viral load measurements were higher for women than for men, and virus was more likely to be undetectable among men than among women, but these differences were of borderline statistical significance. WHO scores could be determined for 70 women and 51 men and were found to be similar for the individuals in the two groups (p = 0.085; Chi-square test). Based on the WHO score, 84.29% of the women and 82.35% of the men were determined to have asymptomatic HIV infection.

**Table 1:** Demographic and Clinical Characteristics for the HIV Seropositive and Seronegative Subjects.

	Seronegative (SN)	Seropositive (SP)	P-value
A. All Patients			
** Number of participants**	**58**	**149**	
** Age (years)**^**1**^	**29.83 (6.41)**	**34.48 (7.58)**	**<0.0001**
** Education (years)**^**1**^	**14.00 (2.41)**	**12.47 (3.27)**	**0.0003**
** Median CD4 count (Q1-Q3)**	**746 (583–898)**	**345 (210–493)**	**<0.0001**
** BDI-II** ^**1**^^,^^**2**^	**5.05 (5.54)**	**7.26 (7.05)**	**0.019**
B. Comparisons by Gender			
Number of participants			
Women	38 (65.52)^2^	92 (61.74)	0.61^3^
Men	20 (34.48)	57 (38.26)	
Age (years)^1^			
Women	30.66 (7.14)	**32.10 (6.43)**	0.26
**Men**	**25.25 (4.49)**	**38.32 (7.77)**	**<0.0001**
p-value (Women vs Men)	0.12	**< .0001**	
Education (years)^1^			
** Women**	**14.24 (2.68)**	**12.70 (2.84)**	**0.005**
** Men**	**13.55 (1.79)**	**12.11 (3.87)**	**0.0296**
p-value (Women vs Men)	0.31	0.32	
BDI-II			
Women	**6.08 (6.10)**	7.60 (6.95)	0.24
** Men**	**3.10 (3.68)**	**6.70 (7.22)**	**0.0058**
p-value (Women vs Men)	**0.025**	0.45	
Median CD4 count (Q1-Q3)			
** Women**	**833.5 (678–977)**	**363 (242.5–508)**	**<0.0001**
** Men**	**631 (469–750.5)**	**265 (171–419)**	**<0.0001**
** p-value (Women vs Men)**	**0.0057**	**0.018**	
C. Viral load			
Log copies/ml			
Women (N = 92)		**4.46 (0.76)**^**1**^	
Men (N = 57)		**4.04 (1.55)**	
p-value		**0.06**	
Non-detectable			
Women (N = 92)		**0/92**	
Men (N = 57)		**3/57**	
p-value^4^		**0.054**	

^1^Mean (Standard Deviation);

^2^Number (Percent);

^3^Chi-square test;

^4^Fisher’s Exact Test. Results that are statistically significant (p<0.05) are in bold letters

### Analysis of Individual Neuropsychological Test Scores for SP versus SN Participants

Analysis of the overall individual neuropsychological test T scores for SN versus SP individuals showed a statistically significant difference only on the Letter (Word Sound) Fluency Test, on which SP individuals performed worse on the test (p = 0.04; Cohen’s d = 0.30). SP individuals also scored lower on the Stroop Color Word Test, but this difference was of borderline significance (p = 0.06; Cohen’s d = 0.29). Performance on the individual neuropsychological tests were also analyzed by gender (Table 2). For this analysis, differences in test mean scores for SP and SN women and men were analyzed by ANOVA with subsequent post-hoc testing for differences between pairs of groups. These studies showed overall differences in performance on the WAIS-III Digit Symbol, WAIS-III Symbol Search, Stroop Color and Word Test, Hopkins Verbal Learning Test-Revised total learning, Brief Visuospatial Memory Test—Revised total learning and with Category Fluency: Verbs (actions). For women in the study, HIV infection was associated with significantly lower scores on the WAIS III Digit Symbol test, WAIS-III Symbol Search, Stroop Color and Word tests, the Hopkins Total Learning Test-Revised total learning and the Brief Visuospatial Memory Test—Revised total learning and Brief Visuospatial Memory Test—Revised delayed recall tests. SP women also scored lower than SN women on the Letter (Word Sound) Fluency and Category Fluency: Nouns (animals) tests, but these differences were of borderline statistical significance. In contrast to what was noted for women in the study, SP men scored significantly better on the Brief Visuospatial Memory Test—Revised total learning test and with Category Fluency: Verbs (actions) than SN men. SP men also performed better than SN men on the finger tapping test, but this difference was of borderline significance. In addition, the effect size values for several tests demonstrated a better performance for SN versus SP men or women on the testing despite the fact that such differences were either not significant or of borderline statistical significance (Table 2).

**Table 2:** Neuropsychological Performance for SN and SP Women and Men.

		Women				Men			
	ANOVA P^1^	SN (N = 38) T Score (SD)	SP (N = 92) T Score (SD)	P	*d*^2^	SN (N = 20) T Score (SD)	(N = 57) T Score (SD	P	*d*
Speed of Information Processing									
** WAIS-III Digit Symbol**	**0.02**	**51.06 (11.25)**	**46.59 (10.46)**	**0.03**	**0.41**	49.91 (11.18)	51.66 (10.69)	0.54	-0.16
** WAIS-III Symbol Search**	**<0.0001**	**51.07 (9.20)**	**46.60 (9.52)**	**0.015**	**0.48**	49.66 (11.60)	54.31 (10.61)	0.10	**-0.42**
Color Trails Test 1 Trial	0.15	48.66 (10.33)	51.37 (11.96)	0.22	**-0.42**	49.67 (9.92)	46.74 (10.76)	0.29	0.28
Trail Making Test A	0.60	50.94 (10.19)	49.31 (12.30)	0.47	0.14	48.65 (9.62)	47.60 (11.32)	0.71	0.10
Attention/Working Memory									
Paced Auditory Serial Addition Task	0.73	48.89 (9.95)	49.57 (9.85)	0.72	**-0.37**	47.91 (13.16)	48.70 (11.76)	0.80	-0.06
WMS-III Spatial Span	0.97	49.98 (10.87)	49.89 (9.44)	0.48	0.01	50.16 (9.05)	50.51 (9.59)	0.89	-0.04
Executive Function									
Color Trails Test 2	0.40	49.72 (10.59)	46.28 (10.61)	0.48	**0.32**	52.58 (10.19)	50.51 (10.07)	0.43	0.20
** Stroop Color and Word Test**	**0.04**	**50.91 (10.86)**	**46.21 (8.72)**	**0.01**	**0.48**	50.03 (8.12)	49.85 (11.95)	0.95	0.02
Learning									
** HVLT-R**^**3**^ **total learning**	**0.009**	**49.50 (11.06)**	**45.03 (9.58)**	**0.02**	**0.43**	48.99 (8.96)	50.15 (9.48)	0.63	-0.13
** BVMT-R**^**4**^ **total learning**	**< .0001**	**50.61 (11.56)**	**46.76 (10.10)**	**0.06**	**0.35**	**48.86 (10.02)**	**54.56 (9.23)**	**0.03**	**-0.59**
Memory									
HVLT-R delayed recall	0.1	49.51 (11.43)	46.57 (9.90)	0.14	**0.36**	48.89 (8.41)	50.49 (9.96)	0.52	-0.17
** BVMT-R delayed recall**	**0.0002**	**51.30 (9.63)**	**46.62 (10.56)**	**0.02**	**0.46**	49.55 (10.62)	54.42 (12.29)	0.12	**-0.42**
Verbal Fluency									
Letter (Word Sound) Fluency	0.20	**50.20 (11.07)**	**46.57 (8.99)**	**0.05**	**0.36**	48.02 (8.80)	46.50 (8.37)	0.49	0.18
Category Fluency: Nouns (animals)	0.29	**51.12 (9.60)**	**47.61 (10.50)**	**0.07**	**0.35**	45.96 (11.71)	49.88 (9.77)	0.15	**-0.33**
** Category Fluency: Verbs (actions)**	**0.0027**	49.68 (9.21)	46.91 (11.43)	0.19	0.27	**47.23 (8.67)**	**53.66 (10.97)**	**0.02**	**-0.65**
Motor Speed and Dexterity									
Grooved Pegboard Test	0.79	49.86 (9.43)	50.63 (11.02)	0.71	-0.08	51.21 (9.26)	51.23 (10.13)	0.99	0.00
Finger tapping test	0.83	51.06 (10.73)	52.07 (14.12)	0.69	-0.08	**48.41 (7.57)**	**52.51 (12.34)**	**0.09**	**-0.40**
Timed gait	0.23	48.47 (9.19)	48.08 (11.10)	0.85	0.04	47.55 (8.19)	44.77 (10.50)	0.29	**0.34**

Comparisons and results that that either have p<0.1 (which is consistent with at least borderline statistical significance) or have at least a moderate effect size (⁥0.3) are in bold letters.

^1^P = p-value.

^2^d = Cohen’s *d*.

^3^Hopkins Verbal Learning Test-Revised.

^4^Brief Visuospatial Memory Test-Revised.

### Analysis of the Ability Domain and Global Deficit Scores for SP versus SN Participants

Mean deficit scores for the seven ability domains and the GDS were compared for all SN and SP individuals (Table 3). SN participants showed better performance with respect to speed of information processing than SP participants. SN subjects also performed better on tests of executive function and motor function, but these differences was of borderline statistical significance. Analysis of global neurocognitive status showed that SN individuals were also overall less impaired, as demonstrated by a mean GDS measure that was lower for SN subjects than for SP individuals. The proportion of all SN and SP individuals who scored in a range that would be considered consistent with NCI were also compared. This analysis showed differences between all SP and all SN participants with respect to speed of information processing and executive functioning, with the latter found to be of borderline statistical significance (Table 3).

**Table 3:** Comparison of ability deficit domain scores and frequency of impairment for all study participants.

Mean Scores	SN	SP	P^1^, *d*^2^
	N, Mean (SD)	N, Mean (SD)	
**SIP**	**58, 0.23 (0.38)**	**148, 0.36 (0.52)**	**0.049, 0.29**
AWM	56, 0.29 (0.62)	146, 0.22 (0.53)	0.47, -0.12
**EF**	**58, 0.20 (0.46)**	**149, 0.35 (0.57)**	**0.075, 0.29**
LN	58, 0.29 (0.58)	149 0.35 (0.57)	0.53, 0.10
MEM	58, 0.27 (0.42)	148, 0.33 (0.59)	0.36, 0.12
VF	58, 0.29 (0.56)	148, 0.34 (0.51)	0.52, 0.09
**MF**	**58, 0.22 (0.30)**	**149, 0.32 (0.42)**	**0.07, 0.27**
**GDS**	**56, 0.25 (0.25)**	**143, 0.34 (0.33)**	**0.047, 0.30**
**% Impaired**	**HIV-**	**HIV+**	**P**
SIP	**6/58**	**39/148**	**0.01**
AWM	7/56	18/146	0.97
EF	**6/58**	**33/149**	**0.05**
LN	8/58	29/149	0.34
MEM	9/58	29/148	0.50
VF	14/58	41/148	0.60
MF	13/58	38/149	0.64
GDS	12/56	39/143	0.40

Comparisons and results that that either have p<0.1 (which is consistent with at least borderline statistical significance) or have at least a moderate effect size (>0.3) are in bold letters.

^1^P = p-value.

^2^d = Cohen’s *d*.

SIP = speed of information processing; AWM = attention/working memory; EF = effector functions; LN = learning; MEM = memory; VF = verbal fluency; MF = motor function; GDS = global deficit score.

Comparison of deficit scores for SP and SN men and women by ANOVA showed differences with respect to learning and memory functions and with respect to the GDS (Table 4). As compared to SN women, SP women had significantly lower mean speed of information processing and verbal fluency scores and lower scores in executive function and memory that were of borderline statistical significance. Also, global performance was better for SN women than for SP women. For men, there was a borderline difference in motor function with SP men performing worse than SN men. Analysis of the frequency of impairment on the testing showed overall differences for speed if information processing, learning and memory for SN and SP men and women (Table 4). Also, SP women were more frequently impaired than SN women on tests of speed of information processing.

**Table 4:** Comparison of ability domain scores and frequency of impairment for women versus men.

		Women			Men		
Mean Scores	ANOVA	SN	SP	P, d^3^	SN	SP	P, d
	(F^1^, P^2^)	N, Mean (SD)	N, Mean (SD)		N, Mean (SD)	N, Mean (SD)	
**SIP**	1.64, 0.18	**38, 0.18 (0.30)**	**92, 0.39 (0.56)**	**0.008, 0.47**	20, 0.31 (0.51)	56, 0.30 (0.47)	0.10, -0.20
AWM	0.41, 0.74	36, 0.29 (0.59)	90, 0.19 (0.45)	0.38, -0.19	20, 0.28 (0.68)	56, 0.27 (0.63)	0.97, -0.20
**EF**	1.23, 0.30	**38, 0.20 (0.46)**	**92, 0.38 (0.61)**	**0.10, 0.33**	20, 0.20 (0.47)	57, 0.30 (0.52)	0.46, 0.20
LN	**3.58, 0.015**	38, 0.32 (0.62)	92 0.46 (0.63)	0.23, 0.21	20, 0.25 (0.50)	57, 0.17 (0.38)	0.44, -0.18
MEM	**4.04, 0.008**	38, 0.28 (0.45)	91, 0.45 (0.67)	**0.09,0.30**	20, 0.25 (0.38)	57, 0.15 (0.38)	0.31, -0.26
VF	1.52, 0.21	**38, 0.22 (0.40)**	**91, 0.40 (0.54)**	**0.04, 0.38**	20, 0.43 (0.77)	57, 0.26 (0.48)	0.36, -0.26
**MF**	1.04, 0.38	38, 0.25 (0.32)	92, 0.31 (0.42)	0.38, 0.16	**20, 0.18 (0.28)**	**57, 0.33 (0.46)**	**0.09, 0.39**
**GDS**	**2.71, 0.046**	**36, 0.23 (0.23)**	**88, 0.38 (0.35)**	**0.0058, 0.51**	20, 0.30 (0.31)	55, 0.27 (0.30)	0.87, -0.10
**% Impaired**	**P**	**HIV-**	**HIV+**	**P**	**HIV-**	**HIV+**	**P**
SIP	**0.02**	**2/38**	**26/92**	**0.004**	4/20	13/56	1.0
AWM	0.64	4/36	9/90	1.0	3/20	9/56	1.0
EF	0.30	4/38	22/92	0.1	2/20	11/57	0.49
LN	**<0.01**	6/38	25/92	0.17	2/20	4/57	0.65
MEM	**<0.04**	6/38	24/91	0.19	3/20	5/57	0.42
VF	0.17^3^	8/38	31/91	0.14	6/20	10/57	0.24
MF	0.96	9/38	22/92	0.98	4/20	16/57	0.57
GDS	0.16	7/36	29/88	0.13	5/20	10/55	0.51

^1^F = F-statistic.

^2^P = p-value.

^3^d = Cohen’s d.

Comparisons and results that either have p<0.1 (which is consistent with at least borderline statistical significance) or have at least a moderate effect size (>0.3) are in bold letters. SIP = Speed of Information Processing; AWM = attention/working memory; EF = effector functions; LN = learning; MEM = memory; VF = verbal fluency; MF = motor function; GDS = global deficit score.

### Analysis of Neuropsychological Test Performance for SP and SN Women and Men by Two-Way ANOVA

Individual neuropsychological test and ability domain deficit scores, GDS and individual neuropsychological test T scores were also compared by gender and serostatus using 2-way ANOVA (Table 5). This analysis showed no differences for SN women and SN men, SN women and SP men or for SP women and SN men (data not shown). Differences were observed, however, on the following tests with scores for SP women that were lower than for SP men: the WAIS-III Symbol Search, the Hopkins Total Learning test, the Brief Visuospatial Memory Test—Revised, the Brief Visuospatial Memory Test—Revised—delayed recall and the Category Fluency: Verbs (actions) test. In addition, SP women had significantly higher learning and memory deficit scores than SP men and, therefore, were more impaired in these domains. There was, however, no difference in the frequency of impairment in the ability domains or with respect to mean GDS (data not shown).

**Table 5:** Differences in Neuropsychological Test Performance and in Ability and Global Deficit Scores for SP Women versus SP Men.

Test or Domain Score Comparison	P^1^, *d*^2^	95% Confidence Interval for the Difference in Scores
**Neuropsychological Test T Score**		
SP Women versus SP Men		
WAIS-III Symbol Search	0.0001, -0.77	-9.93 to -5.14
Hopkins Total Learning	0.03, -0.53	-9.73 to -0.29
Brief Visuospatial Memory Test—Revised	<0.0001, -0.79	-13.03 to -2.98
Brief Visuospatial Memory Test—Revised—Delayed Recall	0.0003, -0.70	-13.33 to -2.72
Category Fluency: Verbs (actions)	0.004, -0.59	-11.79 to -1.41
**Ability Domain Deficit Score**		
Learning	0.018, 0.80	0.03 to 0.57
Memory	0.01, 0.57	0.05 to 0.57

^1^P = p-value.

^2^d = Cohen’s *d*.

### Comparison of Monocyte, sCD14 and sCD163 Measures for HIV SP and SN Individuals

**Fig 1:**
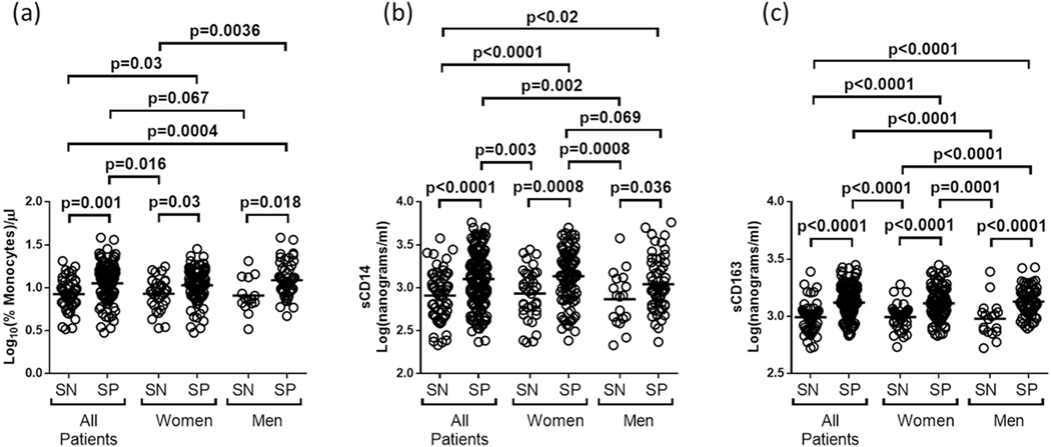
Comparison of monocyte, sCD14 and sCD163 measurements. A comparison was performed of (a) log_10_(%monocyte), (b) sCD14 and (c) sCD163 measurements for all SN and SP subjects and for SN and SP women and men. In nearly all cases, significant differences between SN and SP individuals overall and between the female and male gender groups. Similar levels of these measures were observed for the various SP groups (see the text for additional details).

Numbers of circulating monocytes and plasma levels of sCD14 and sCD16 were compared for all HIV SP and SN participants and for SP and SN women and men in the cohort (Fig 1). These studies showed that absolute monocyte numbers were overall higher for SP than for SN individuals. Also, circulating monocyte numbers for SP women and SP men were higher than those for their SN counterparts and numbers for SP participants overall and for SP men were also higher than those for SN individuals overall and higher than for SN women.

For sCD14, levels were also overall higher for SP than for SN individuals and higher for SP women than for SN women and for SP men then for SN men. Other comparisons showed that levels for nearly all SP groups were higher than those for any of the SN groups. Also, SP women had sCD14 levels that were higher than SP men, but this difference was a borderline statistical significance. For sCD163, measurements for all SP individuals were higher than for those in any of the SN groups.

### Correlations between GDS and Monocyte, sCD14 and sCD163 Measures

Correlations between GDS, monocyte numbers, sCD14, sCD163 and numbers of CD4+ T cells were examined for all SN and SP participants and for the gender and HIV serostatus subgroups (Table 6A). No correlation was observed between any of these measures among SN subjects overall or for SN women. GDS correlated with sCD14 and, at a borderline level of significance, with sCD163 levels among all SP participants, with only sCD14 levels among SP women and, at a borderline level of significance, with sCD163 among SN men. Monocyte numbers correlated inversely with CD4+ T cell counts among all SP groups as well as among SN men.

**Table 6:** Correlations (p-values) between Clinical Laboratory Parameters of HIV Infection and Measures of Monocyte Activation by Subject HIV Serostatus and Gender.

**A. Correlations for All SP and SN Participants**					
	**GDS**	**Monocytes**	**sCD14**	**sCD163**	**CD4 Count**
**All SN**					
GDS		0.048 (0.75)	0.003 (0.98)	0.216 (0.11)	0.184 (0.17)
Monocytes	0.048 (0.75)		0.206 (0.16)	0.085 (0.56)	-0.165 (0.26)
sCD14	0.003 (0.98)	0.206 (0.16)		0.0116 (0.91)	-0.017 (0.90)
sCD163	0.216 (0.11)	0.085 (0.56)	0.016 (0.91)		-0.014 (0.92)
CD4 Count	0.184 (0.17)	-0.165 (0.26)	-0.017 (0.90)	-0.014 (0.92)	
**All SP**					
GDS		-0.062 (0.48)	**0.212 (0.011)**	**0.153 (0.069)**	-0.038 (0.66)
Monocytes	-0.062 (0.48)		1.74 E-6 (1.0)	0.116 (0.17)	**-0.329 (<0.0001)**
sCD14	**0.212 (0.011)**	1.74 E-6 (1.0)		0.089 (0.28)	-0.136 (1.0)
sCD163	**0.153 (0.069)**	0.116 (0.17)	0.089 (0.28)		-0.125 (0.13)
CD4 Count	-0.038 (0.67)	**-0.329 (<0.0001)**	-0.136 (1.0)	-0.125 (0.13)	
**SN Women**					
GDS		0.150 (0.43)	-0.006 (0.97)	0.007 (0.97)	0.277 (0.10)
Monocytes	0.150 (0.43)		0.113 (0.54)	0.199 (0.28)	-0.070 (0.71)
sCD14	-0.006 (0.97)	0.113 (0.54)		0.079 (0.64)	0.115 (0.49)
sCD163	0.007 (0.97)	0.199 (0.28)	0.079 (0.64)		0.027 (0.87)
CD4 Count	0.277 (0.10)	-0.070 (0.71)	0.115 (0.49)	0.027 (0.87)	
**SP Women**					
GDS		0.019 (0.87)	**0.366 (<0.0001)**	0.176 (0.10)	-0.153 (0.15)
Monocytes	0.019 (0.87)		-0.063 (0.56)	0.155 (0.15)	**-0.330 (0.002)**
sCD14	**0.366 (<0.0001)**	-0.063 (0.56)		0.069 (0.51)	-0.10 (0.35)
sCD163	0.176 (0.10)	0.155 (0.15)	0.069 (0.51)		**-0.203 (0.05)**
CD4 Count	-0.153 (0.15)	**-0.330 (0.002)**	-0.100 (0.35)	**-0.203 (0.05)**	
**SN Men**					
GDS		-0.357 (0.46)	0.012 (0.96)	**0.447 (0.063)**	0.142 (0.58)
Monocytes	-0.357 (0.19)		0.318 (0.25)	-0.258 (0.35)	**-0.542 (0.037)**
sCD14	0.012 (0.96)	0.318 (0.31)		-0.055 (0.83)	-0.265 (0.29)
sCD163	**0.447 (0.063)**	-0.258 (0.37)	-0.055 (0.83)		-0.159 (0.53)
CD4 Count	0.142 (0.58)	**-0.542 (0.037)**	-0.265 (0.28)	-0.159 (0.53)	
**SP Men**					
GDS		-0.143 (0.32)	-0.057 (0.68)	0.083(0.54)	0.078 (0.56)
Monocytes	-0.143 (0.32)		0.094 (0.51)	0.132 (0.35)	**-0.277 (0.047)**
sCD14	-0.057 (0.68)	0.094 (0.51)		0.145 (0.27)	-0.176 (0.18)
sCD163	0.083 (0.54)	0.132 (0.35)	0.145 (0.27)		-0.077 (0.56)
CD4 Count	0.078 (0.56)	**-0.277 (0.047)**	-0.176 (0.18)	-0.077 (0.56)	
B. Correlations (p-values) with Viral Load					
	**GDS**	**Monocytes**	**sCD14**	**sCD163**	**CD4 Count**
**All SP**	**0.222 (0.008)**	**0.219 (0.01)**	**0.174 (0.034)**	**0.220 (0.007)**	**-0.388 (<0.0001)**
**SP Women**	**0.191 (0.075)**	**0.330 (0.002)**	**0.266 (0.01)**	**0.264 (0.01)**	**-0.554 (<0.0001)**
**SP Men**	**0.241 (0.077)**	0.176 (0.22)	0.138 (0.31)	0.176 (0.19)	**-0.381 (0.0035)**

Results that are of at least borderline statistical significance (p<0.1) are in bold letters.

For SP individuals, correlations with HIV viral load were also examined (Table 6B). These studies showed, for all groups, significant inverse correlations for CD4+ T cell counts and viral load measurements. For GDS, a significant correlation with viral load was observed for the entire group of SP individuals and borderline statistically significant correlations for SP women and SP men. Among the entire group of SP individuals and for SP women, significant correlations were observed between viral load and percent monocyte, sCD14 and sCD163 measurements; however, no significant correlations were observed between viral load and these measures for SP men.

## Discussion

In this study we report results that confirm our previous findings demonstrating the occurrence of neurocognitive impairment among a cohort of individuals in Nigeria with HIV infection [6]. We also demonstrate the presence of increased monocyte numbers and, as reported for other cohorts [12–14,22,23], elevated levels of monocyte activation among infected individuals. A surprising result was the finding that individuals who demonstrated impairment on the individual neuropsychological tests and within the specific ability domains were primarily women. Furthermore, in a multivariate analysis we showed that the differences that were observed were specifically between SP women and SP men. Results similar to these findings, as well as the observation that SP men also performed better than SN men on the testing, were obtained by Hested et al in studies involving a smaller cohort in Zambia [7]. In two other previously reported studies, HIV-infected women were also more likely than men to present with neurologic symptoms and evidence of neurologic disability [24] and to progress more rapidly to AIDS despite having similar viral plasma load measurements [25]. The latter study, however, involved individuals who were illicit drug users, and such individuals were excluded from our Nigeria cohort. Also, the finding that gender can impact the rate of progression of neurological disease or time to the development of AIDS has not been replicated in other studies [26,27].

The reasons for our observations involving SP women in our study are unclear, and the findings are particularly striking given the fact that the SP women were younger and had higher CD4+ T cell counts than their male counterparts, factors that are generally associated with a better performance on the neuropsychological testing [28,29]. Also, BDI-II scores were similar for SP women, SN women and SP men, and in the subclinical range. Therefore, the reason for the poor overall performance by SP women on the testing cannot be explained by the presence of symptoms of depression. Previous studies of gender effects on performance on the specific neuropsychological tests that were included in the study battery show that women may perform better on the Stroop test with respect to color naming and word reading and on the Grooved Pegboard test [30]. Men, on the other hand, tend to perform better on the Finger-tapping test [30]. This notwithstanding, tests scores in our study were adjusted for demographic effects based on the performances of the SN group. Among the other factors that could underlie the presence of impairment among SP women include hormonal effects, which may modulate immune responses to either increase or decrease protective immunity in HIV infection. In this regard, it has been observed that estrogens and progestin may increase immune reactivity to antigens, the expression of immune mediators and circulating CD4+ T cell numbers, with such effects changing at the different phases of the menstrual cycle [31]. Other possibilities are that differences may exist in the availability of healthcare to the women who we enrolled into the study or that socio-cultural, economic (lower income), educational or other factors may exist for the women that could predispose them to developing neurocognitive impairment or not performing as well on the tests. Such factors could also explain why on several tests SP men performed better than SN men.

HIV enters the central nervous system early in infection, where it is likely transported by infected monocytes [32]. Once in the nervous system, toxicity is induced by direct effects from HIV products and inflammatory mediators that are secreted by activated phagocytes in the brain [33]. Among the SP individuals and SN men in our cohort, peripheral blood monocyte numbers and markers of monocyte activation were found to be elevated and showed an inverse correlation with CD4+ T cell counts. This has been previously described and is likely a reflection of the relative lymphopenia that occurs as a result of HIV infection [34]. The reason for this observation among SN men is unclear. Other factors that can impact circulating monocyte numbers in men include increased serum cholesterol levels, which can decrease numbers of monocytes and cigarette smoking [35,36]. In both men and women monocyte numbers can be increased by exercise [37]. None of these factors were examined as a part of this study, however. We also found that levels of the measured monocyte activation markers correlated with severity of global impairment. This is consistent with several studies that have demonstrated that the presence and severity of neurocognitive impairment among infected individuals correlate with levels of sCD14 and sCD163 in plasma [12,13]. In the studies that we report here, not only did we show that sCD14 and sCD163 levels are increased among HIV SP individuals in the cohort, but we also provide the first demonstration of increased numbers of blood monocytes among infected individuals in association with an increase in the levels of these activation markers. In addition, sCD14 levels were slightly higher for SP women than for SP men, which is consistent with the finding that SP women performed worse on the neuropsychological testing than SP men. CD14, the endotoxin receptor, is produced by monocytes and macrophages and, in binding lipopolysaccharide, uses Toll receptor 4 (TLR4) as a co-receptor. Soluble CD14 is secreted by the cells following activation and increased levels of sCD14 occur in association with elevated levels of lipopolysaccharide and, in HIV infection, with microbial translocation [38] and the presence of neurocognitive impairment [12–14,22,23].

CD163 is a hemoglobin scavenger receptor that is also produced by monocytes and macrophages [39–43]. Like sCD14, levels of sCD163 are increased by LPS, and this effect has been demonstrated to occur in vitro and in vivo [41,42,44]. In vitro sCD163 can inhibit the proliferation of T cells in culture [45], and, therefore, may have immunosuppressive effects in vivo that could promote earlier development of more advanced disease. In normal and in HIV-1-infected brain, CD163 is expressed by perivascular macrophages that are likely derived from CD14+CD16+CD163+ monocytes circulating in peripheral blood [46]. In studies performed by Fitch et al it was demonstrated plasma levels of sCD163 are higher in women than in men [47]. However, the patients in this study were older than our study participants, were on treatment with antiretroviral drugs, and their neurocognitive status was not assessed [47]. Blood CD16+ monocytes may be also more likely to be infected with HIV-1 [48], and in a study performed during the pre-ART era, it was found that numbers of CD14+CD16+ monocyte are elevated in individuals with HIV-associated dementia [17], which is associated with decreased survival [49]. Elevated sCD14 levels have been linked with an increased mortality [16,50], and, the same is likely for sCD163, which is associated with an increased risk of development of HIV-associated dementia [49,51],

HIV viral load measures were associated with lower CD4+ T cell counts among all participant groups. Viral load measures were also slightly higher for women than for men, and women were less likely to have non-detectable virus than men. Viral load levels correlated with global impairment for the cohort overall but showed a borderline correlation with GDS for both women and men, likely as a result of the smaller numbers of individuals in the subgroups. For the activation markers, correlations for sCD14 and sCD163 with viral load were noted for only women in the study. These findings further highlight the likelihood that mechanisms that underlie the development of HIV-related neurocognitive impairment differ for women and men.

## Conclusion

In conclusion, SP women in our Nigerian cohort performed worse than SP men on a number of neuropsychological tests, representing several cognitive domains. These findings among women were associated with a borderline higher HIV viral burden; however, the viral load measures correlated with levels of the measured immune markers. Global impairment in women was associated with elevated levels of one of these markers, sCD14. Such gender-specific findings have not been previously demonstrated in any HIV-infected cohort. Our ability to observe such differences may be due to the fact that most study participants had clinically asymptomatic HIV infection. With that passage of time and the development of HIV-related symptoms and initiation of antiretroviral therapy, the differences will likely become more difficult to identify. There are several other important limitations to this study. These include the relatively small overall sample size and the fact that the group sizes were unbalanced for SN versus SP participants in general and for women versus men. Such limitations could have influenced the outcome of specific analyses. For example, the relatively small number of SN may have resulted in decreased precision in the calculation and application of demographic adjustments. This is likely reflected in the borderline level of statistical significance found for some analyses, particularly comparisons involving data from men, who comprised just slightly over one third of all individuals in the cohort. Therefore, the results that we report here will need to be confirmed in future analyses of data from a larger number of study participants. It is also possible that the findings may have been influenced by an inadvertent selection bias that resulted from the fact that participants were recruited from counseling and testing centers located within the two hospitals in Abuja. In this context, it is possible that men may avoid hospital settings as places to seek HIV testing and counseling. It is also possible that, due to sociocultural issues, the neuropsychological test battery was limited in its ability to detect abnormalities equally in men and women. However, for areas of the world where resources are limited, information such as that which is described in this report could provide an opportunity for early intervention and to otherwise provide care to infected individuals at a time when the disease burden may be lower. Doing so may offer the potential to improve clinical outcomes for individuals following the initiation of treatment and to potentially decrease the overall expense of care.
